# Case report: Microwave ablation is a safe and effective method for primary hyperparathyroidism in pregnancy

**DOI:** 10.3389/fmed.2024.1204696

**Published:** 2024-01-17

**Authors:** Yunbo Luo, Qi Lv, Zhou Xu, Jiang Fang, Hongyu Pu, Yanchun Gao, Shuangqiang Qian, Fei Chen, Xiaobo Zhao, Lingmi Hou

**Affiliations:** ^1^Department of Thyroid and Breast Surgery, Affiliated Hospital of North Sichuan Medical College, Nanchong, China; ^2^Department of Operating Room, Affiliated Hospital of North Sichuan Medical College, Nanchong, China; ^3^Department of Nuclear Medicine, Affiliated Hospital of North Sichuan Medical College, Nanchong, China; ^4^Department of Academician (Expert) Workstation, Biological Targeting Laboratory of Breast Cancer, Breast and Thyroid Surgery, Affiliated Hospital of North Sichuan Medical College, Nanchong, China

**Keywords:** primary hyperparathyroidism, parathyroid adenoma, hypercalcemia, microwave ablation, pregnancy

## Abstract

Primary hyperparathyroidism (PHPT) is a rare disease in pregnancy and endangers the health of both pregnant women and fetuses. However, the treatments are very limited for PHPT and most of them are unsatisfactory because of the peculiar state in pregnancy. The only curable method is parathyroidectomy which can be safely performed in the second trimester of pregnancy. In this case, we reported a pregnant woman with primary parathyroid adenoma presenting hypercalcemia and severe vomit at the end of first trimester. Finally, she got cured by microwave ablation at the end of first trimester and gave birth to a healthy baby boy.

## Introduction

Primary hyperparathyroidism (PHPT) is often characterized by hypercalcemia and can lead to bone loss, kidney stones, digestive disease and cardiovascular disease (1–3). PHPT is more likely to appear in women than men and its incidence increases with age (4, 5). The incidence of PHPT is very low during pregnancy, with less than 1% in previous studies (6–8). However, the latest research revealed that eight patients (2.1%) had PHPT in a cohort including 386 Indian pregnancy women (9). PHPT is a severe disease in pregnancy, which may lead to maternal and fetal life-threatening complications. It can cause hyperemesis gravidarum, nephrolithiasis and pancreatitis in pregnant women (10–12), and it can also lead to hypocalcemia, tetany, intrauterine growth retardation and fetal demise in fetus (8, 13, 14). Due to the rarity of this disease among pregnant women and the special state of pregnancy, the treatments for PHPT are very limited and the only curable option is parathyroidectomy (13, 15, 16). But parathyroidectomy can only be safely performed in the second trimester and is not suitable for the first or third trimesters. In recent years, several thermal ablation techniques, including high intensity focused ultrasound (HIFU), radiofrequency ablation (RFA), microwave ablation (MWA), and laser ablation (LA), have been used as alternatives to parathyroidectomy in non-pregnant patients, with the cure rate of 81–92% for PHPT (17–20). Theoretically, the above thermal ablation techniques can also be applied to pregnant women with PHPT. Therefore, we reported a case that was cured by MWA at the end of first trimester.

## Case presentation

### Clinical presentation and diagnosis of the patient

**Figure 1:**
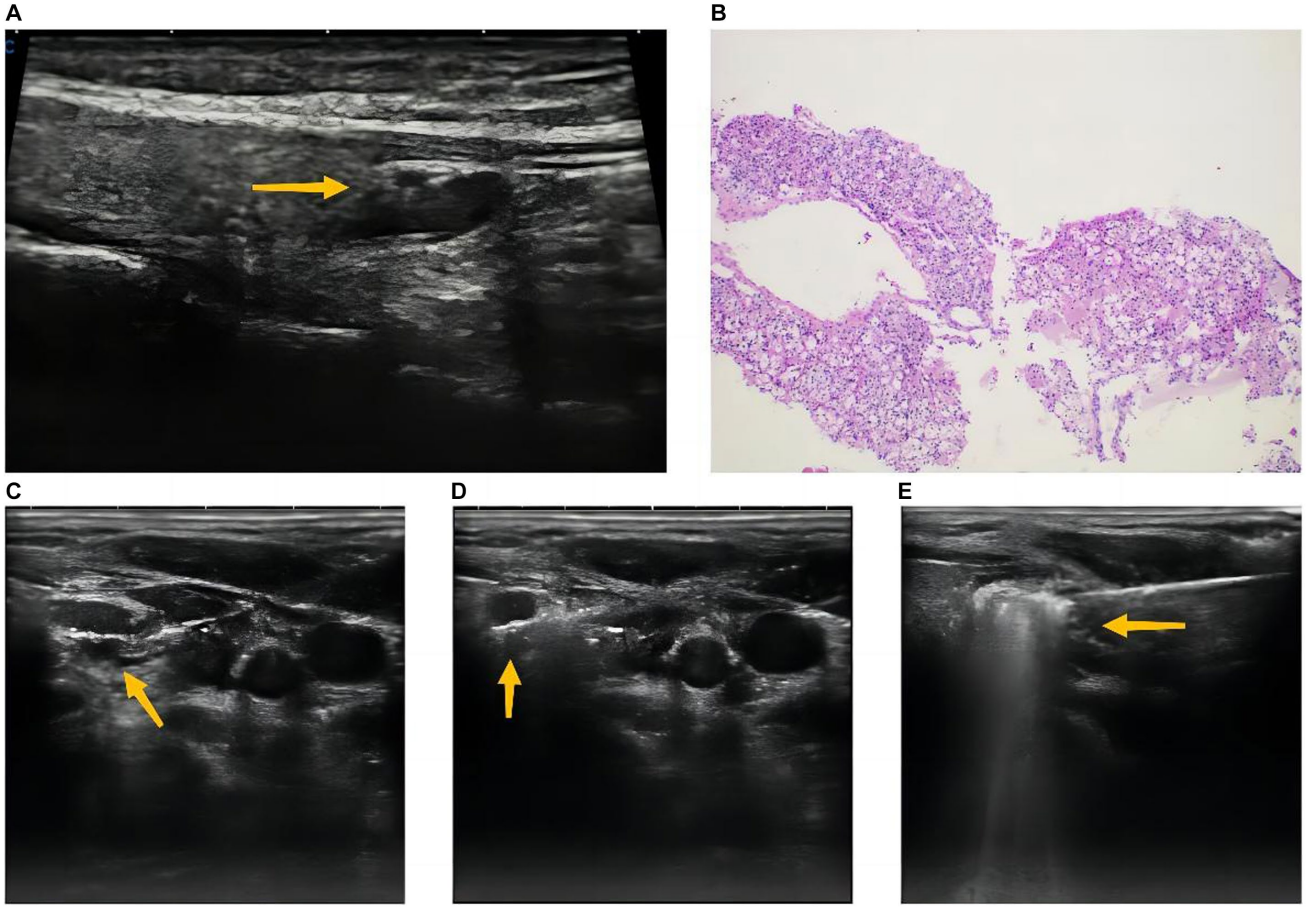
Ultrasound revealing a hypoechoic area with 1.1*0.3 cm on the dorsal side of the left inferior pole of thyroid **(A)**. Parathyroid adenoma was confirmed by core needle biopsy, which was composed of chief, transitional, oncocytic, and water clear cells **(B)**. Normal saline solution was injected into the region between the parathyroid adenoma and vital structures of the neck to protect recurrent laryngeal nerve, esophagus and trachea adjacent to the parathyroid adenoma **(C)**. Antenna was located in the parathyroid adenoma **(D)**. Ablation was finished until the entire nodule was covered with hyperechoic microbubbles **(E)**.

A 21-year-old pregnant woman presented with severe nausea and vomiting for 1 month and was admitted to obstetrics department when she was 12 weeks pregnant. Although hyperemesis gravidarum is the most common symptom of early pregnancy, blood electrolyte test was performed to determine whether electrolyte disorders existed. Unexpectedly, the result showed hypercalcemia with serum calcium of 3.4 mmol/L (reference range, 2.11–2.52 mmol/L). Then, further test showed serum intact parathormone (iPTH) with 128 pg./mL (reference range, 18.5–88 pg./mL). The ultrasound revealed a hypoechoic area with 1.1*0.3 cm on the dorsal side of the left inferior pole of thyroid (Figure 1A), which is a common location for lower polar parathyroid. Besides, this hypoechoic area lacked lymph node structures, such as cortex or medulla of lymph node, which would rule out the central compartment lymph node. Therefore, the hypoechoic area was considered to be a parathyroid adenoma because of hypercalcemia and abnormal parathyroid hormone levels. Computed tomography (CT) and ^99m^Tc-methoxyisobutylisonitrile (^99m^Tc-MIBI) scanning were not applied to this patient for the potential risks to fetus. Core needle biopsy (18-gauge) was used to obtain more tissue sample because of relatively large parathyroid lesion and easy location. Finally, it was confirmed to be parathyroid adenoma by routine sectioning and staining with hematoxylin and eosin (Figure 1B). Considering patient’s young age, we had informed the patient about the necessity of genetic testing to rule out the multiple endocrine neoplasia, such as MEN1, MEN2, MEN4, etc. However, due to the absence of family history of MEN, the patient refused further genetic testing to identify the potential hereditary disease. We have informed the patient to pay close attention to the possibility of MEN in the follow-up procedure.

### Treatment process

Conservative approaches, including low-calcium diet and oral fluid rehydration were suggested for this patient. Also, the patient received intravenous fluid rehydration: in the first 24 h, 150 mL saline per hour; then, 125 mL saline per hour for 5 days (21). However, the level of serum calcium decreased slightly and maintained in the range of 3.1–3.2 mmol/L. The symptoms of nausea and vomiting did not relieve as well. Parathyroidectomy became the only effective treatment for this pregnant woman. While, the patient really wanted to have this fetus and parathyroidectomy might cause miscarriage or malformation of the fetus at the first trimester. Consequently, the women refused the parathyroidectomy and requested a safer treatment. Continuation of conservative treatment might endanger the lives of pregnant woman and her fetus because of hypercalcemia. Finally, microwave ablation of parathyroid adenoma became a possible solution and approved by the patient.

The microwave ablation system (XR-A1408W, Nanjing Great Wall Medical Equipment Co, Nanjing, China) was used and consisted of a microwave generator producing 30–35 W of power at 2450 MHz either continuously or in a pulse, a flexible cable and an internally-cooled 16-gauge thyroid antenna with 8 cm shaft length and a 1.4 mm active tip. The ablation area around the tip is an ellipse, which can be adjusted according to the ablation time and power. In this case, we planned to continue the ablation for 20 s with 30 W of power, which could ablate the area with 12.3*6.7 mm. The patient was in supine position with mild hyperextension of her neck before the microwave ablation was performed and was continuously monitored in the whole process. After determining the optimal puncture location, local anesthesia was performed with 2% lidocaine. Then, normal saline solution was injected into the region between the parathyroid adenoma and vital structures of the neck to protect recurrent laryngeal nerve, esophagus and trachea adjacent to the parathyroid adenoma (Figure 1C). The antenna was inserted into the parathyroid adenoma while avoiding the blood vessels under ultrasound guidance. After determining that the tip of the needle was located in parathyroid adenoma (Figure 1D), ablation was started under closely ultrasound monitoring. Moving the needle tip to ablate the whole parathyroid adenoma until the entire nodule was covered with hyperechoic microbubbles (Figure 1E). The whole process of ablation was very eventful and no complications happened to this patient. More than twenty-minute of cold compression was applied to this patient after the ablation to reduce the risk of neck hematoma.

## Results

**Figure 2:**
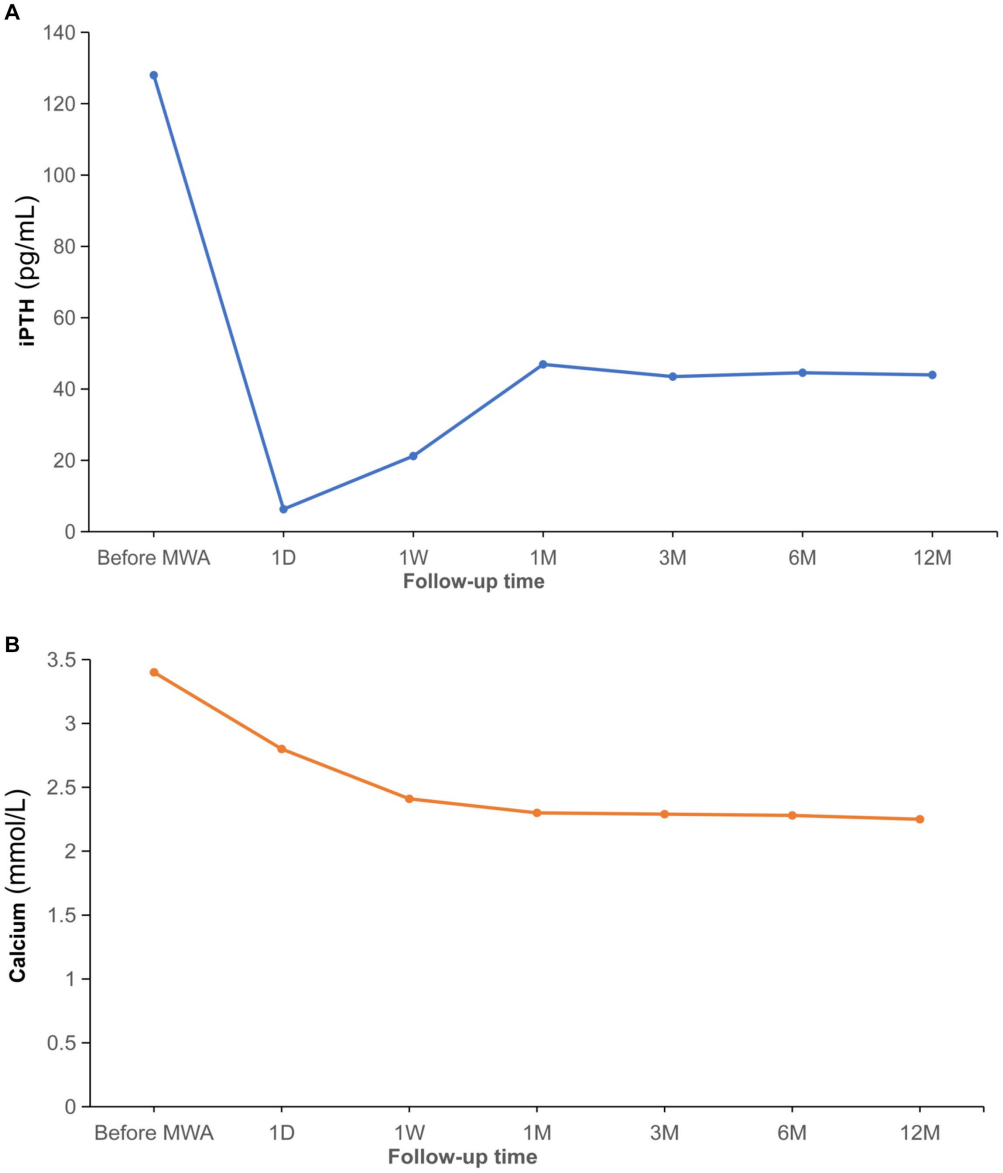
Comparations before and after ablation for iPTH **(A)**. Comparations before and after ablation for serum calcium **(B)**. iPTH, intact parathormone; MWA, microwave ablation; D, day; W, week; M, month.

**Figure 3:**
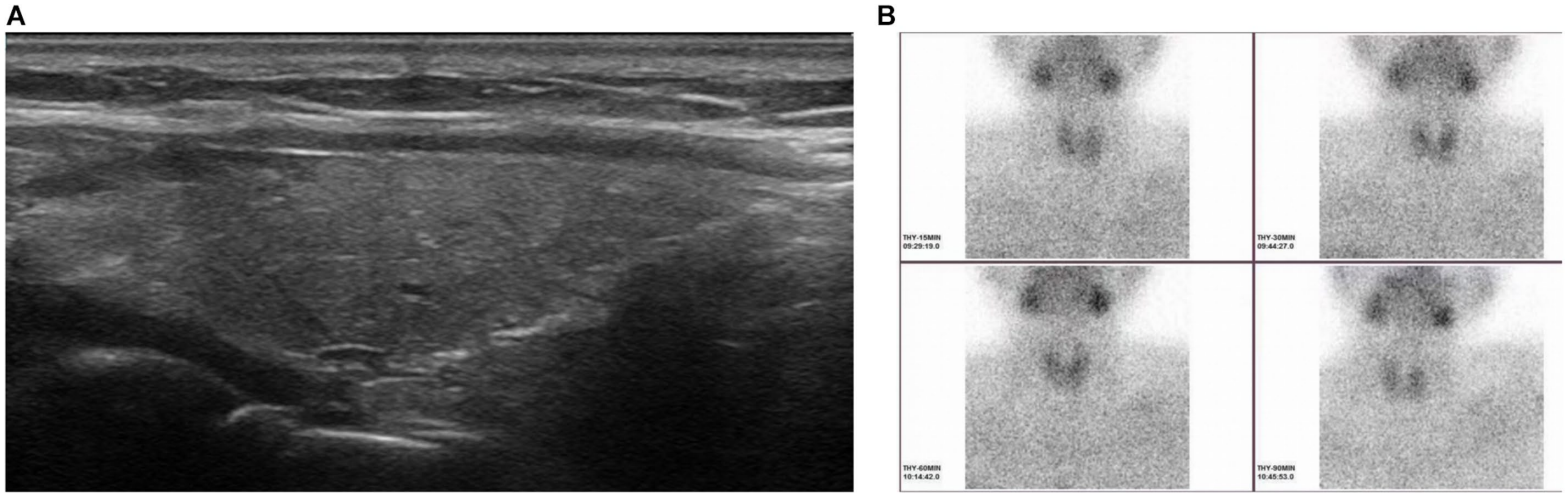
Ultrasound showing the primary hypoechoic area (parathyroid adenoma) disappeared 1 year after microwave ablation **(A)**. No hyperplastic parathyroid was found in both the early and delayed phases (15, 30 and 60 min after the injection of methoxyisobutylisonitrile) when ^99m^Tc-methoxyisobutylisonitrile was performed 1 year after microwave ablation **(B)**.

Clinical data, including the levels of serum iPTH and calcium, were collected in the subsequent follow-up (1 day, 1 week, 1 month, 3 months, 6 months and 12 months after ablation). As shown in Figure 2, the levels of serum iPTH and calcium decreased to 10.3 pg./mL and 2.8 mmol/L after 1 day. And the symptoms of nausea and vomiting released significantly as well. After 1 week, the levels of serum iPTH and calcium stayed within normal range (21.2 pg./mL and 2.41 mmol/L, respectively). After 1 month, the level of serum iPTH slightly increased to 46.9 pg./mL, but it still belonged to normal range. The level of serum calcium remained within normal range, with 2.3 mmol/L. After 3 months, the levels of serum iPTH and calcium remained stable (43.48 pg./mL and 2.29 mmol/L, respectively). The rest of the pregnancy went uneventfully and a healthy baby boy was born by cesarean section half 1 year after ablation. The tests demonstrated that both of mother and her baby had normal serum calcium (2.35 and 2.31 mmol/L, respectively). The iPTH and calcium of the patient were remaining within normal ranges during follow-up (6 months, 43.48 pg./mL and 2.28 mmol/L; 12 months: 43.95 pg./mL and 2.25 mmol/L). One year later, it was surprised that ultrasound revealed the primary hypoechoic area (parathyroid adenoma) disappeared (Figure 3A). Furthermore, ^99m^Tc-methoxyisobutylisonitrile (^99m^Tc-MIBI) confirmed no hyperplastic parathyroid in both the early and delayed phases (Figure 3B).

## Discussion

Primary hyperparathyroidism (PHPT) in pregnancy is a rare disease, but it may cause great complications for both pregnant women and fetuses. Hyperemesis gravidarum and nephrolithiasis are the most common complications for pregnant women (7, 9, 12). In addition, acute pancreatitis and preeclampsia are the most serious complications (7, 9, 12, 22), which may lead to the death of pregnant women. Previous studies have reported that PHPT can easily cause neonatal hypocalcemia (22–24). Other complications, including polyhydramnios, neonatal hypocalcemia, intrauterine growth retardation, premature delivery and fetal death, have also been reported (22, 25, 26). Therefore, early diagnosis and effective treatments are crucial for pregnant women with PHPT.

More than 50% of patients were asymptomatic and diagnosed with PTHT accidentally in pregnancy (13). Therefore, the diagnosis of PHPT in pregnancy mainly relies on the presence of an elevated serum ionized calcium and an elevated iPTH during routine pregnancy examination. Parathyroid adenoma is the most common cause of PTHT, parathyroid hyperplasia occurs less frequently and parathyroid carcinoma is extremely rare found causing PTHT. Besides, hereditary disorders may occur in 10% of patients, and those patients often present PTHT at young age and have familial parathyroid disorders (27). Accurate localization of the above lesion is very crucial for successful treatment, but most of the imaging methods are restricted in pregnancy. Neck ultrasound is the safest approach and also has high sensitivity and accuracy in localizing parathyroid adenoma by a skilled operator (28). In addition, magnetic resonance imaging (MRI) without contrast can also be applied to the diagnosis and localization for parathyroid adenoma (12). Computed tomography (CT) and ^99m^Tc-MIBI scanning should be avoided for the potential risks of fetal malformations or death, intellectual deficiency, or subsequent cancer from the ionizing radiation (29, 30). Apart from the imaging methods mentioned above, fine-needle biopsy with iPTH-washout concentration is a reliable method to diagnose and localize the parathyroid lesions (31, 32). Also, core needle biopsy could be used to obtain more tissue sample and observe the lesion by routine sectioning and staining with hematoxylin and eosin when the parathyroid lesions are relatively large and well positioned. However, there are certain risks associated with any invasive procedures, such as neck hematoma and damages to the recurrent laryngeal nerve, esophagus and trachea. Besides, parathyromatosis is also a serious complication after needle biopsy of parathyroid lesions (33). Therefore, we must inform the patients of the risks associated with needle biopsy and take measures to avoid them as much as possible.

Due to the rarity of PTHT during pregnancy, there is no consensus available for the treatment of PTHT in pregnancy. Thus, individualized treatment is required for pregnant women with PHPT depending on the severity of symptoms, gestational age, desire of fertility and complications. Conservative management maybe suitable for patients with asymptomatic and mild PHPT (serum calcium <2.85 mmol/L). A low-calcium diet combining with adequate hydration, including oral or intravenous fluids, are the main approaches for reducing serum calcium. However, such conservative approaches have no significant effectiveness in most cases, especially in pregnant women with severe symptoms (12, 34, 35). Bisphosphonates and calcitonin are effective drugs for non-pregnant patients with PTHT, but bisphosphonates may be toxic to embryo and calcitonin often causes tachyphylaxis (36). Cinacalcet is categorized as class C in pregnancy because of its ability to cross the placenta and lacking long-term safety data in pregnancy. Three out of six cases were diagnosed with neonatal hypocalcemia after treated with cinacalcet (37–39). Therefore, the cinacalcet should be used with great caution in pregnant women. Parathyroidectomy is a curable way for most cases and can be safely performed in the second trimester. A retrospective study including 17 cases with gestational PHPT showed that parathyroidectomy was successfully performed in 14 patients (82.4%) during the second trimester (13). In addition, a systematic review showed that 108 patients (28.3%) underwent parathyroidectomy during pregnancy (16). Among the patients getting surgery, 67.7% of the patients chose parathyroidectomy in the second trimester and complications occurred in 4.48% of patients. Unfortunately, surgery-related complications and/or deaths happened to 25% of patients in the first trimester and 21.1% of patients in the third trimester. Therefore, parathyroidectomy is more suitable for patients with gestational PHPT in the second trimester but not for patients in other trimesters. In our case, the patient was at the end of first trimester and parathyroidectomy might cause serious complications for both pregnant woman and fetus, which made medical decisions very difficult. Numerous researches have revealed that microwave ablation is an efficient therapy for primary hyperparathyroidism and has few complications in non-gestational patients (19, 40–42). Fortunately, US-guided microwave ablation has been very successfully performed in our case and no complication happened to the pregnant woman and fetus. Besides, the symptoms of nausea and vomiting got released when the levels of serum iPTH and calcium returned to normal ranges. Finally, she gave birth to a healthy baby boy and maintained a normal level of serum calcium during subsequent follow-up. Microwave ablation is an efficient thermal ablation technique for primary hyperparathyroidism and can be performed at any time during pregnancy theoretically. It causes few complications and has cosmetic advantage because of the minimally invasive technique. In addition to microwave ablation, other ablation methods may be also effective for PHPT. A case report showed that a patient with PHPT presented severe hypercalcemia and acute pancreatitis in pregnancy and got cured by ultrasound-guided ethanol ablation of parathyroid adenoma (12). However, as reported in previous researches (43, 44), ethanol ablation is often unsuccessful at once and may need twice or more repetitive performance for PHPT. Thus, ultrasound-guided microwave ablation maybe a better choice among the above ablative approaches. Nevertheless, because the above ablative techniques are not current standard therapy for PHPT, microwave ablation maybe advocated when parathyroidectomy is refused or not feasible.

## Conclusion

PHPT is a rare disease in pregnancy and has huge potential risks to both pregnancy women and fetuses. Ultrasound-guided microwave ablation is a safe and effective approach for PHPT at early stage of pregnancy.

## Data availability statement

The original contributions presented in the study are included in the article/supplementary material, further inquiries can be directed to the corresponding authors.

## Ethics statement

The studies involving humans were approved by Ethics Committee of Affiliated Hospital of North Sichuan Medical College. The studies were conducted in accordance with the local legislation and institutional requirements. The participants provided their written informed consent to participate in this study. Written informed consent was obtained from the individual(s) for the publication of any potentially identifiable images or data included in this article. Written informed consent was obtained from the participant/patient(s) for the publication of this case report.

## Author contributions

YL, ZX, and LH conceived and designed this study and reviewed the manuscript. YL, QL, ZX, HP, JF, and SQ processed the pictures and organized the manuscript. YG, QL, and FC completed the microwave ablation treatment and took images. All authors contributed to the article and approved the submitted version.
